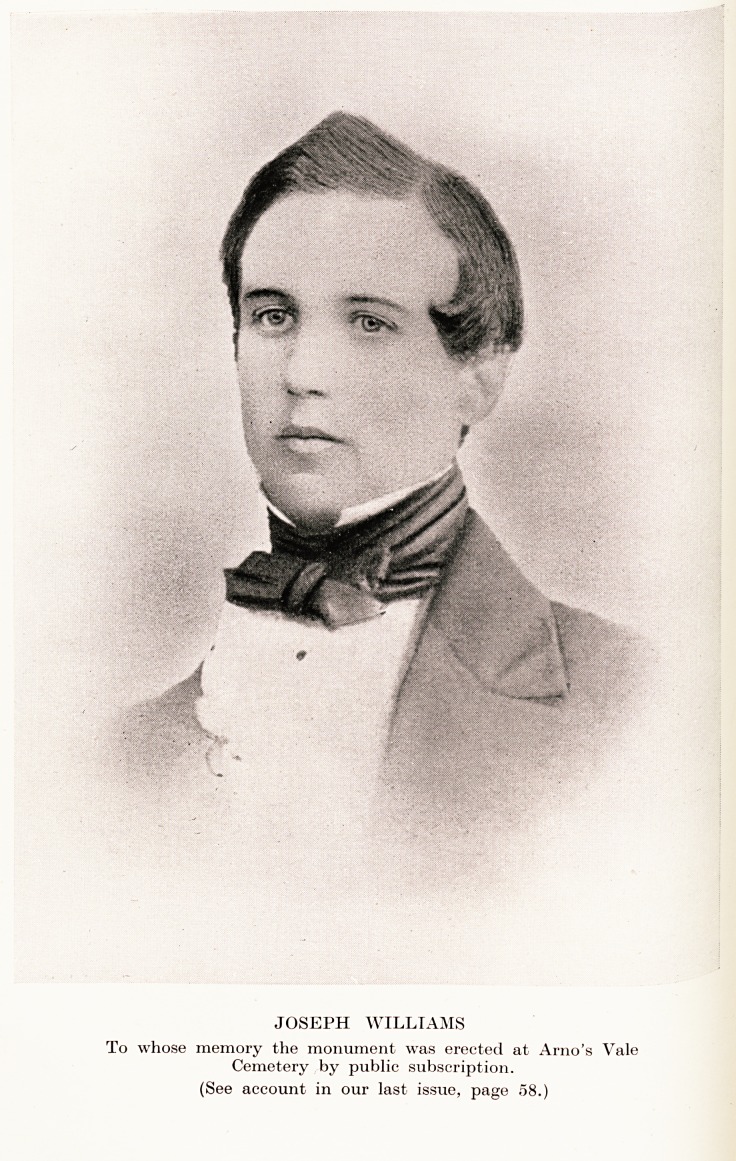# Joseph Williams

**Published:** 1936

**Authors:** 


JOSEPH WILLIAMS

To whose memory the monument was erected at Arno's Vale Cemetery by public subscription.

(See account in our last issue, page 58.)

## Figures and Tables

**Figure f1:**